# Complex posttraumatic stress disorder in intergenerational trauma transmission among Eritrean asylum-seeking mother-child dyads

**DOI:** 10.1080/20008066.2023.2300588

**Published:** 2024-01-08

**Authors:** Rahel Bachem, Yafit Levin, Kim Yuval, Nora Korin Langer, Zahava Solomon, Amit Bernstein

**Affiliations:** a Psychopathology and Clinical Intervention, Department of Psychology, University of Zurich, Zurich, Switzerland; b School of Social Work, Ariel University, Ariel, Israel; c Observing Minds Lab, Department of Psychology, School of Psychological Sciences, University of Haifa, Haifa, Israel; d Bob Shapell School of Social Work, Tel-Aviv University, Tel-Aviv, Israel; e the I-Core Research Center for Mass Trauma, Tel-Aviv, Israel; f Observing Minds Lab, Department of Psychology, School of Psychological Sciences, University of Haifa, Haifa, Israel

**Keywords:** Asylum-seekers, child socio-emotional development, complex posttraumatic stress disorder, depression, intergenerational trauma transmission, Solicitante de asilo, desarrollo infantil socioemocional, trastorno de estrés postraumático complejo, depresión, transmisión intergeneracional del trauma

## Abstract

**Background:** Traumatic stress among forcibly displaced people has a variety of adverse consequences beyond individual mental health, including implications for poor socioemotional developmental outcomes for their children post-displacement.

**Objective:** This study explored the intergenerational transmission of maternal ICD-11 Complex Posttraumatic Stress Disorder (CPTSD) and depression among asylum-seeking mothers for their children’s internalizing and externalizing difficulties.

**Method:** Participants were 127 trauma-affected Eritrean mothers of preschool-aged children in Israel. The severity of child difficulties was compared between mothers with probable ICD-11 CPTSD (94.5% comorbid depression), ICD-11 PTSD (48.5% comorbid depression), unimorbid depression, and healthy mothers, using multivariate analyses of variance, while controlling for children’s direct exposure to adverse life experiences.

**Results:** Probable ICD-11 CPTSD and PTSD were present in 23.6% and 26.0% of mothers, respectively. Relative to maternal PTSD, CPTSD was significantly and strongly associated with elevated child internalizing symptoms (*d* = 2.44) and marginally significantly, although strongly, associated with child externalizing symptoms (*d* = 1.30). Post-hoc exploratory analyses documented that, relative to maternal PTSD and depression, CPTSD and depression comorbidity was marginally significantly but strongly associated with child internalizing (SMD = .67), but not externalizing symptoms (SMD = .35).

**Conclusions:** Findings implicate maternal CPTSD and comorbid depression in child socio-emotional development and inform clinical assessment, prevention, and intervention to attenuate poor development among children in unstable post-displacement settings.

## HIGHLIGHTS

- Trauma among forcibly displaced people has a variety of aversive multisystemic consequences, compromising the socioemotional development of non-exposed children.
- ICD-11 complex posttraumatic stress disorder (CPTSD) and comorbid depression may be functionally important to elevated risk for maternal intergenerational trauma transmission, even relative to ICD-11 posttraumatic stress disorder (PTSD).
- To effectively attenuate intergenerational transmission of trauma post-displacement, efforts and resources should be invested in maternal mental health care as well as socio-culturally adapted, trauma-sensitive parenting training.

## Abstract (es)

**Antecedentes:** El estrés traumático entre las personas desplazadas a la fuerza tiene una variedad de consecuencias adversas más allá de la salud mental individual, incluyendo pobres resultados en el desarrollo socioemocional de sus hijos después del desplazamiento.

**Objetivo:** Este estudio exploró la transmisión intergeneracional del trastorno de estrés postraumático complejo (TEPT-C) materno, según la CIE-11, y la depresión materna de las madres solicitantes de asilo por las dificultades internalizantes y externalizantes de sus hijos.

**Método:** Las participantes fueron 127 madres eritreas de niños en edad preescolar viviendo en Israel afectadas por un trauma. Se comparó la gravedad de las dificultades de los niños de madres con probable TEPT-C según la CIE-11 (con un 94,5% de depresión comórbida) con aquellas con diagnósticos de TEPT según la CIE-11 (con un 48,5% de depresión comórbida), de depresión sin comorbilidad y con madres sanas, utilizando análisis de varianza multivariados mientras se controlaba la exposición directa de los niños a experiencias vitales adversas.

**Resultados:** Se encontraron casos probables de TEPT-C y de TEPT en el 23.,6% y el 26% de las madres, respectivamente. En relación con el TEPT materno, el TEPT-C se asoció de forma significativa y fuerte con síntomas internalizantes infantiles elevados (*d* = 2.44). Asimismo, se asoció de manera marginalmente significativa, aunque fuerte, con síntomas externalizantes infantiles (*d* = 1.30). En relación con el TEPT y la depresión materna, los análisis exploratorios post hoc documentaron que la comorbilidad del TEPT-C con la depresión fue marginalmente significativa, pero fuertemente asociada con síntomas infantiles internalizantes (SMD = .67), mas no con síntomas externalizantes (SMD = .35).

**Conclusiones:** Los hallazgos relacionan el TEPT-C y la depresión comórbida en las madres con el desarrollo socioemocional de los hijos y brindan información para que la evaluación clínica, prevención e intervención atenúen el pobre desarrollo de los niños en entornos inestables luego de desplazamientos forzados.

Forcibly displaced people, including refugees and asylum-seekers, are among the most severely burdened populations in terms of traumatic stress exposure followed by chronic stress exposure post-displacement (de Silva et al., 2021). They often experience a myriad of adverse and potentially traumatic events in the pre-, peri- and post-migration context, which include armed conflict, loss or separation from family and friends, life-threatening journeys to receiving countries, lengthy asylum processes, and complex demands on acculturation in the host communities (Hou et al., 2020). Thus, refugees and asylum-seekers are at elevated risk for stress-related mental health problems, most notably post-traumatic stress disorder (PTSD) (e.g. Blackmore et al., 2020). Although studied less extensively, traumatic stress outcomes of forced displacement have a variety of destructive multi-systemic outcomes beyond individual mental health, including the intergenerational transmission of trauma and stress to refugee children’s socioemotional development post-displacement.

In post-displacement settings, children of traumatized refugees and asylum-seekers are exposed to intergenerational trauma transmission, which refers to the impact of trauma-related distress, experienced by a parent, on child development and wellbeing (van Ee et al., 2012). For example, research using different diagnostic conceptualizations (DSM-IV, DSM-5, ICD-10) has implicated PTSD symptoms in poor parent-child interactions (e.g. characterized by irritability, hostility, anxiety) and social learning (e.g. children observing fearfulness, avoidant, or withdrawal reactions) within families (Lang & Gartstein, 2018; van Ee et al., 2016). Importantly, parental depressive symptoms have also been implicated in the intergenerational transmission of trauma, manifesting particularly in child internalizing symptoms (Goodman, 2020; Roubinov et al., 2022).

A limited number of studies have investigated intergenerational trauma transmission among refugees and asylum-seekers. Two systematic reviews documented intergenerational trauma transmission among refugee families, whereby higher levels of parental trauma exposure and trauma-related distress were associated with poor child mental health and well-being, including child posttraumatic stress symptoms, depressive symptoms, anxiety, attention deficiency, and psychosocial stress (Flanagan et al., 2020; Sangalang & Vang, 2017). Flanagan et al. (2020) reviewed eight studies that focused on intergenerational trauma transmission in refugee and asylum-seeking families with children and adolescents born post-migration. Likely mechanisms of transmission identified in the reviewed studies include insecure attachment, maladaptive parenting styles, diminished maternal emotional availability, diminished family functioning, and dysfunctional intra-family communication styles. Yet, despite important progress, significant questions regarding intergenerational trauma transmission among refugee and asylum-seeker families remain unanswered.

Although parental stress- and trauma-related mental health are implicated in child socioemotional outcomes, no study has sought to disentangle the intergenerational effects of ICD-11 PTSD and CPTSD on child outcomes. The ICD-11 introduced a differentiated conceptualization of stress-related disorders with a hierarchical distinction between a narrower diagnosis of PTSD characterized by fear reactions (i.e. re-experiencing the traumatic event(s), avoidance of trauma-related cues, and a sense of current threat manifested by excessive hypervigilance or an enhanced startle reaction) and a broader diagnosis of Complex PTSD (CPTSD) characterized by these fear reactions as well as additional disturbances in self-organization (DSO; WHO, 2018) (i.e. affective dysregulation, a negative self-concept, and disturbances in relationships). CPTSD is typically associated with multiple, prolonged experiences of interpersonal traumatization from which it is difficult to escape (Maercker et al., 2022) and which are often encountered by refugees and asylum-seekers (Frost et al., 2019). Indeed, CPTSD prevalence in adult refugee and asylum-seeker samples ranged from 2.2% to 50.9%, depending on the geographic location, traumatic exposure, and post-migration setting (de Silva et al., 2021). These observations may be critical to better understanding intergenerational transmission of stress and trauma among refugee and asylum-seeker families. Indeed, in other populations, it was reported that CPTSD, relative to PTSD, was related to higher levels of functional impairment in the areas of family and relationship problems (Karatzias et al., 2017) and more attachment anxiety and avoidance (e.g. Karatzias et al., 2022). Yet, we do not know whether CPTSD and DSO symptoms, relative to the narrower PTSD symptoms, may be particularly significant in the intergenerational transmission of stress and trauma to children of trauma-affected refugees and asylum-seekers in post-displacement settings. The implications of this understanding may not only be crucial for significantly advancing the understanding of intergenerational transmission of trauma for child developmental trajectories among forcibly displaced people but, clinically, for prevention as well as specialized intervention development and delivery.

In addition to CPTSD and PTSD, a meta-analysis has also documented an elevated prevalence of depression (30% to 40%) among refugees and asylum-seekers (Henkelmann et al., 2020). Moreover, studies have documented elevated comorbidity between depression and PTSD (e.g. Haselgruber et al., 2021) and even greater rates of comorbidity between depression and CPTSD symptoms (e.g. Hyland, Shevlin, Elklit, et al., 2017). This is important because maternal depression has long been linked to poor child mental health outcomes in a variety of populations including refugees and asylum-seekers (e.g. East et al., 2018). Yet, we do not know what role, if any, depression may play in the association between ICD-11 CPTSD and DSO symptoms, relative to the narrower ICD-11 PTSD symptoms, for intergenerational transmission of stress and trauma to children of trauma-affected refugees and asylum-seekers. The implications of the role of depression in the context of posttraumatic stress may be critical to not only better understand intergenerational trauma transmission for child developmental trajectories among forcibly displaced people but, clinically, for prevention and specialized intervention development and delivery.

Finally, to advance understanding of intergenerational transmission of stress and trauma among refugees and asylum-seekers, it is essential to dissociate the effect of often co-occurring aversive life events among children post-displacement from intergenerational transmission of parental trauma. Several studies pointing to parental intergenerational transmission of trauma and stress among refugees and asylum-seekers have not accounted for children’s direct exposure to adverse or potentially traumatic experiences post-displacement, making it difficult to ascribe poor child socioemotional developmental outcomes to parental intergenerational transmission per se (Sangalang & Vang, 2017). This is particularly important to this area of work because many post-displacement settings not only contribute to elevated risk for poor maternal or parental mental health but also increase the risk for adverse childhood experiences (Rizkalla et al., 2020).

Thus, the first objective of this study was to examine whether children of mothers with ICD-11 CPTSD, PTSD or without a stress-related disorder display different levels of internalizing and externalizing problems while controlling for children's direct exposure to adverse life events. The second objective was to better understand the contribution of depression in the context of ICD-11 CPTSD and PTSD, by comparing child internalizing and externalizing problems among children of mothers with CPTSD vs. PTSD vs. depression but no PTSD vs. healthy mothers (no CPTSD, PTSD, or depression). We hypothesized that probable maternal ICD-11 CPTSD would be most strongly associated with elevated child internalizing and externalizing symptoms.

## 1. Method

### 1.1. Participants

The current study focused on a high-risk sample of asylum-seeking Eritrean mothers and their preschool-aged children in Israel. Israel has experienced a surge of asylum-seeking refugees who predominantly arrived from Eritrea and Sudan (Nakash et al., 2017). While crossing through the Sinai, many migrants fell prey to human traffickers and passed through so-called torture camps before they finally arrived at the Israeli border in a highly traumatized state. Moreover, Israeli authorities have denied this population stable residential status and, consequently, less than 0.5% have received refugee status. In this way, government policies deprive asylum-seekers of social security benefits, health insurance and other social services and contribute to a highly unstable postmigration setting (ASSAF, 2022; Nakash et al., 2017).

### 1.2. Procedure

A sample of Eritrean mothers of preschoolers seeking asylum in Israel was recruited. Inclusion criteria were being female, able to answer questions in the Eritrean national language (Tigrinya) with the help of a female Eritrean research assistant and having a preschool-aged child. Data were collected from May to August 2019 in Tel Aviv, Israel. Recruitment was conducted in collaboration with a local NGO (www.unitaf.org), which offers nurseries, daycare, and after-school programmes for asylum-seeking and migrant children. By operating a community-based model of preschool programmes, Unitaf provides a safe environment for 1300 children with liminal legality each year. Eritrean project assistants approached mothers at the unitaf centres and invited them to data collection in groups of two to eight women. A Ph.D. level psychologist (RB) and two trained research assistants provided standardized instructions and explanations or administered the questionnaire as a structured interview (e.g. due to illiteracy of some participants). The study was approved by the Ethics IRB Committee of Tel Aviv University and participants were made aware that participation was entirely voluntary. Participants gave written informed consent after receiving explanations of the forms in Tigrinya. Study materials were presented in Tigrinya and English. Mothers provided information about themselves and the oldest of their preschool-aged children. Completing the assessment battery took on average 2.5 h and participants were compensated with 120 NIS (approximately 35 Dollars).

A total of 128 dyads of Eritrean mothers and children participated in this study. One participant could not complete the questionnaire due to emotional distress during the assessment, resulting in a final sample of 127 participants. Mother’s age ranged from 19 to 42 years (*M* = 28.47, *SD* = 3.95), and children’s age ranged from 2 to 6 years (*M* = 4.31, *SD* = .97). Most mothers (95.3%) were born in Eritrea, 4.7% were Tigrinya-speaking persons born in other countries such as Sudan. The children’s gender distributed almost equally with 51.2% girls and 48.8% boys. They immigrated to Israel between June 2007 and December 2013. All children were born post-migration in Israel and thus did not share maternal pre- and peri-migration trauma exposure. The demographic characteristics of the sample are depicted in Table 1.

**Table 1.** Sample demographics.

	*M*	*SD*	*N*	%
*Mothers*				
Age	28.47	3.95		
Marital status				
Married			106	84.8
Separated / divorced			15	12.0
Never married			3	2.4
Widowed			1	0.8
Registered partnership			0	0
Number of children	2.56	1.04		
Years of education	8.20	3.03		
< 5			13	10.2
5–8			58	45.7
9–12			53	41.7
> 12			3	2.4
Employment status				
Full time (Min 30 h/week)			51	40.8
Part-time (less than 30 h/week)			22	17.6
Day-worker (work varies week-to-week)			22	17.3
Does not work, currently searching			18	14.4
Does not work, not currently searching			12	9.6
*Children*				
Age	4.31	.97		
Gender				
Girls			63	51.2
Boys			60	48.8

Note. *N* = 127 dyads of mothers and children.

### 1.3. Measures

#### 1.3.1. Translation

All measures were first translated from English to Tigrinya by an Eritrean translator experienced in quantitative psychological research on posttraumatic consequences among forcibly displaced Eritreans and the translation of psychometric questionnaires for similar research projects as the current one. Then, they were back-translated to English in a blind, written form by a second, independent Eritrean translator. Discrepancies in wording and grammar between the original version and back-translation were identified and consequently discussed by authors (RB, KY) and both translators, until a consensus was reached based on the the guidelines Brislin (1970) and Geisinger (1994) offer. The translated questionnaires, of which only a subset was examined in the present project, were pilot-tested to ensure their acceptability for the study population, and minor revisions were made to ensure comprehension by the target population.

#### 1.3.2. Maternal trauma exposure

Lifetime exposure to potentially traumatizing events with the 17-item trauma checklist of the Harvard Trauma Questionnaire (HTQ; Mollica et al., 1992). This checklist has been used to assess trauma in refugee populations and was found to be reliable and valid in multiple studies with traumatized refugees (Hollifield et al., 2002). The HTQ assesses trauma exposure on four levels: (1 = not experienced; 2 = heard about; 3 = witnessed; 4 = experienced). To represent trauma exposure, we computed a sum score of the number of traumatic event types (possible range: 17–68).

#### 1.3.3. Maternal PTSD and CPTSD

The International Trauma Questionnaire (ITQ; Cloitre et al., 2018) is a self-report instrument to assess ICD-11 PTSD and CPTSD. It assesses the severity of PTSD symptoms in the past month: re-experiencing (two items), avoidance (two items), and sense of current threat (two items) on a five-point Likert scale (0 = not at all, 4 = very strong), as well as DSO symptoms related to affect dysregulation (two items), negative self-concept (two items), and disturbed relationships (two items). The five-point Likert scale assessed to which degree the symptoms typically feel, think, or relate to others (from 0 = not at all to 4 = very strong). The continuous scores of ICD-11 PTSD severity and ICD-11 CPTSD DSO severity were computed for all mothers. Additionally, three items (0 = not at all, 4 = very strong) were used to assess functional impairment through PTSD and DSO, respectively. The ITQ was found to be a reliable instrument in diverse refugee populations (Vallières et al., 2018) and internal consistency in this study was satisfactory (Cronbach's α = .860) for PTSD and excellent (Cronbach's α = .940) for DSO.

Diagnostic criteria for ICD-11 PTSD and CPTSD were operationalized following a standardized ITQ scoring algorithm, corresponding to the ICD-11 diagnostic criteria: Scores ≥2 (‘moderately’) indicated the categorical presence of a diagnostic symptom. A probable diagnosis of PTSD requires endorsement of one symptom from the symptom clusters of (1) re-experiencing, (2) avoidance, and (3) sense of current threat, plus the endorsement of at least one indicator of functional impairment. Endorsement of a symptom or functional impairment is defined as a score ≥ 2. A diagnosis of CPTSD requires the endorsement of one of two symptoms from each of the three PTSD symptoms clusters and one symptom from each of the DSO clusters: (1) affective dysregulation, (2) negative self-concept, and (3) disturbances in relationships. Functional impairment was identified if at least one indicator of functional impairment was endorsed related to PTSD symptoms and one indicator was endorsed related to DSO symptoms (Cloitre et al., 2018).

#### 1.3.4. Maternal depression symptoms

The nine-item Patient Health Questionnaire-9 (PHQ-9; Kroenke et al., 2001) assesses the severity of DSM-IV major depressive disorder during the preceding two weeks. Each item of PHQ-9 was scored on a scale of 0–3 (0 = not at all; 3 = nearly every day). The PHQ-9 total score ranges from 0 to 27 (scores of 5–9 are classified as mild depression; 10–14 as moderate depression; 15–19 as moderately severe depression; ≥ 20 as severe depression) (Spitzer et al., 2014). The two latter categories were used as indicators of probable depression in the current sample. The PHQ-9 has been previously studied among East African refugees (e.g. Aizik-Reebs et al., 2021) and showed satisfactory internal consistency in this study (Cronbach's α = .88).

#### 1.3.5. Child emotional and behavioural difficulties

The 100-item Child Behavior Checklist (CBCL; Achenbach & Rescorla, 2000) for ages 1.5–5 years lists internalizing and externalizing child difficulties and asks parents to indicate how well each item fits their child, using a scale ranging from 0 (not true) to 2 (very true or often true). The measure has strong psychometric properties (Achenbach & Rescorla, 2000). Internalizing difficulties include syndrome scores of emotionally reactive, anxious/depressed, somatic complaints, sleep problems and withdrawal, while externalizing difficulties reflect syndrome scores of aggressive behaviour and attention problems. Internal consistencies in the current study were .94 and .95 for the internalizing and externalizing difficulties, respectively.

#### 1.3.6. Adverse child experiences

Assessment of children’s adverse experiences was based on the Traumatic Events Screening Inventory–Parent Report (TESI-PRR; Ippen et al., 2002), assessing potentially traumatic events among children up to 6 years. Items in the current study included 12 adverse experiences assessed via a dichotomous format (0 = not experienced; 1 = experienced): death in the family or someone close to the child, severe accident, hospitalization of the child or a close family member, separation from parents, depression of a close family member, physical abuse, emotional abuse, sexual abuse, arrest or imprisonment of family member, alcohol or drug addiction of close family member, exposure to fierce quarrels or domestic violence as well as an open question of any other significant child experience.

### 1.4. Data analysis

G*Power (Version 3.1) was used to conduct a power analysis for MANCOVA using power 1-β = .8, α = .05 and an effect size (f² = .10), which is considered small, with three groups and two dependent variables. The analysis informed that a sample size of *n* = 88 and *df* = 192 is needed to detect a global main effect. For the 4-groups comparisons with all other indicators similar, a sample size of *n *= 116 and *df* = 224 is needed to detect a global main effect. A sensitivity power analysis with the resulting sample size of *n* = 120 at power 1-β = .8 and α = .05 allowed detection of an effect as small as ηp² = 0.070.

Data were analyzed with IBM SPSS Statistics Version 25.0. Missing item-level data ranged between 3.1% and 10.2%. Little’s Missing Completely at Random test (MCAR) revealed that the data were missing at random (χ²(325) = 36.92, *p* = 1.00). First, Pearson correlations of PTSD, DSO, and depression with the child's difficulties were conducted. Then, categorical scores were calculated from ITQ and PHQ to increase the meaningfulness of the results in clinical settings, where diagnosis is also commonly used in a dichotomous way and to account for the fact that CPTSD is likely not only the simple sum of PTSD and DSO symptoms but a different condition due to their possible interaction (Cloitre et al., 2018). Next, we tested for stress-related disorders group differences in the child's difficulties using multivariate analyses of variance with covariate (MANCOVA) with externalizing and internalizing difficulties of the child as the dependent factors, controlling for the child's life events. Specifically, three groups were compared: children of mothers with probable ICD-11 CPTSD, children of mothers with probable ICD-11 PTSD, and children of mothers without probable ICD-11 CPTSD or PTSD. Cohen's d is reported for all comparisons due to the small group sizes. By convention, Cohen's *d* of .2, .5, .8 are considered small, medium and large effect sizes, respectively (Cohen, 1988).

To explore the role of depression and its shared morbidity with stress-related disorders, we examined the differences in externalizing and internalizing difficulties of the children between four groups of mothers: (1) with probable PTSD, (2) with probable CPTSD, (3) with depression only (no PTSD or CPTSD), and (4) with no probable depression, CPTSD or PTSD. Here, we aimed to inspect all comparisons as well as additional specific contrasts that complemented the main analysis: a comparison of child outcomes between mothers with CPTSD and depression vs. mothers with PTSD and depression, for which effect size was determined by standardized mean difference (SMD) for contrasts. The latter comparison was intended to shed light on the role of CPTSD above and beyond depression. In the MANOVA, we used Pillai's trace test statistic which gives more robust results in the case of homogeneous variance (Ates et al., 2019). The test statistics used with MANOVA are affected by the violation of homogeneity of covariance matrices and normality assumptions particularly from an unbalanced number of observations, as observed in the current study.

## 2. Results

### 2.1. Descriptive results

As shown in Table 2, trauma exposure of the participating mothers was high. The most prevalent potentially traumatic event was lack of food or water, experienced by *n* = 118 (93.7%). However, interpersonal trauma, such as torture (*n* = 70, 55.1%) or sexual abuse (*n* = 23 18.1%) was also frequently indicated. Regarding mental health, 13 mothers (10.2%) reported mild depression; 35 (27.6%) moderate depression; 36 (28.3%) moderately severe depression, and 43 (33.9%) severe depression. Thirty-three mothers (26.0%) fulfilled the criteria for probable ICD-11 PTSD, among whom 16 (48.5%) also had probable depression; 30 (23.6%) fulfilled the criteria for probable ICD-11 CPTSD, among whom 28 (94.5%) reached criteria for probable depression, and 64 (50.4%) mothers had no probable diagnosis of ICD-11 PTSD or CPTSD. There were 35 (27.6%) mothers with depression only and 29 (22.8%) mothers who did not reach the criteria for (C)PTSD or depression. Correlations, means and standard deviations of the study's variables are presented in Table 3. The four diagnosis groups did not differ significantly concerning children’s age F(4, 116) = 1.14 *p* = .341, mother’s age F (4, 119) = .63 *p* = .641, mother’s employment F (4, 120) = .410 *p* = .801, and mothers’ education F (4, 122) = 2.31 *p* = .062.

**Table 2.** Descriptive results trauma exposure.

	Not experienced	Heard about	Witnessed	Experienced
	*n* (%)			
Lack of food or water	3 (2.4)	4 (3.1)	1 (0.8)	118 (93.7)
Ill health without but no medical care	20 (15.7%)	13 (10.2)	30 (23.6)	62 (48.8)
Lack of shelter	9 (7.1)	17 (13.4)	7 (5.5)	91 (71.7)
Imprisonment	20 (15.7)	38 (29.9)	10 (7.9)	58 (45.7)
Serious injury	30 (23.6)	25 (19.7)	25 (19.7)	46 (36.2)
Combat situation	19 (15.0)	33 (26.0)	25 (19.7)	48 (37.8)
Brain washing	35 (27.6)	27 (21.3)	8 (6.3)	56 (44.1)
Rape or sexual abuse	36 (28.3)	50 (39.4)	16 (12.6)	23 (18.1)
Forced isolation from others	25 (19.7)	24 (18.9)	7 (5.5)	70 (55.1)
Being close to death	21 (16.5)	15 (11.8)	31 (24.4)	59 (46.5)
Forced separation from family	20 (15.7)	23 (18.1)	8 (6.3)	74 (58.3)
Murder of a family member or friend	27 (21.3)	47 (37.0)	23 (18.1)	29 (22.8)
Unnatural death of family / friend	26 (20.5)	44 (34.6)	25 (19.7)	30 (23.6)
Murder of a stranger or strangers	21 (16.5)	61 (48.0)	25 (19.7)	18 (14.2)
Being lost or kidnapped	12 (9.4)	48 (38.6)	25 (19.7)	40 (31.5)
Torture	13 (10.2)	26 (20.5)	16 (12.6)	70 (55.1)
Any other situation	1 (0.8)	81 (63.8)	6 (4.7)	29 (22.8)

**Table 3.** Intercorrelations of study variables.

	Traumatic events mother	PTSD mother	DSO mother	Depression	Adverse child experience	CBCL internalizing	CBCL externalizing
Traumatic events mother	*1*	* *	* *	* *	* *	* *	* *
PTSD mothers	.554**	*1*	* *	* *	* *	* *	* *
DSO mother	.417**	.586***	*1*	* *	* *	* *	* *
Depression	.480***	.580***	.803***	*1*	* *	* *	* *
Adverse child experiences	.194*	.263**	.310**	.426***	*1*	* *	* *
CBCL internalizing	.217*	.429**	.447**	.317**	.317**	*1*	* *
CBCL externalizing	.193*	.339**	.359**	.236*	.236*	.753**	*1*
*M*	47.71	12.09	10.39	18.72	1.50	20.11	14.94
*SD*	11.00	5.80	7.47	7.31	1.72	14.25	9.09

Note. CBCL = Child Behavior Checklist; CBCL internalizing = CBCL internalizing difficulties; CBCL externalizing = CBCL externalizing difficulties. *p < .05, **p < .01, ***p < .001.

### 2.2. Maternal stress-related group differences in child outcomes

We first set out to test the primary question regarding child socioemotional outcomes associated with ICD-11 CPTSD relative to ICD-11 PTSD. Using *Pillai's* criterion for MANOVA, the multivariate dependent variable of child difficulties – externalizing and internalizing difficulties – was significantly different between the three groups, F (4, 190) = 3.32, *p* = .012, partial η^2^ = .11, observed power = .871 (Table 4). Post-hoc univariate analysis showed that children of mothers with CPTSD had significantly more internalizing difficulties relative to children of mothers with PTSD (Cohen’s *d* = 2.44) as well as mothers without CPTSD or PTSD (Cohen’s *d* = 2.44). The effects were of a large magnitude. Importantly, on internalizing difficulties, children of mothers with PTSD did not differ significantly from children of mothers without either CPTSD or PTSD (some of whom did have depression; Cohen’s *d* = .08).

**Table 4.** Child difficulties in the context of maternal stress-related disorders.

	No stress-related diagnosis *n *= 64 (50.4%)	PTSD *n* = 33 (26%)	CPTSD *n* = 30 (23.6%)	Probable Diagnosis	*p*	Life events	*p*
** **				F (2, 95)		F (1, 95)	
CBCL Internalizing	16.78 (12.47)	16.42 (8.37)	28.96 (18.35)	5.67**	.005	5.91*	.017
CBCL Externalizing	12.48 (8.20)	14.42 (7.57)	19.92 (10.78)	4.70*	.011	3.19	.077

Note. PTSD = Posttraumatic stress disorder, CPTSD = complex posttraumatic stress disorder. CBCL internalizing = CBCL internalizing difficulties; CBCL externalizing = CBCL externalizing difficulties. *p < .05, **p < .01.

For child externalizing difficulties, a different pattern of differences was observed. Children of mothers with CPTSD had significantly greater externalizing difficulties than children of mothers without CPTSD or PTSD (Cohen’s *d* = 2.06). A marginally significant difference, which was large in magnitude, was also observed between children of mothers with CPTSD and children of mothers with PTSD (Cohen’s *d* = 1.30). However, no significant difference (Cohen’s *d* = 0.54) was observed between children of mothers with PTSD and children of mothers without CPTSD or PTSD. The same pattern of results was observed in analyses using continuous scores of CPTSD and PTSD, as well as when comparing CPTSD DSO symptomatology more specifically and PTSD, above and beyond the child’s adversities (see Supplementary Materials).

### 2.3. Maternal depression and stress-related group differences in child outcomes

Due to very high comorbidity between CPTSD and depression, we sought to test the secondary question related to the role of depression in observed differences between ICD-11 CPTSD and ICD-11 PTSD. In contrast to the model above, here we compared internalizing and externalizing child outcomes among mothers with CPTSD (most of whom also had depression), mothers with PTSD (some of whom also had depression), mothers with depression only, and healthy mothers (without CPTSD, PTSD, or depression), again using *Pillai’s* criterion for MANOVA. The difference in the global score was significant between the groups, *F* (6,200) = 3.39, *p* = .004, partial η2 = .18, observed power = .921. Examination of each dependent variable separately showed differences in both externalizing and internalizing difficulties (Table 5).

**Table 5.** Child difficulties in the context of maternal stress-related disorders and depression.

	No stress-related disorder or depression *n* = 22 (22.2%)	Depression only *n* = 28 (28.3%)	PTSD *n* = 24 (24.2%)	CPTSD *n* = 25 (25.3%)	Probable Diagnosis	*p*	Life events	*p*
					F (3,100)		F (1, 95)	
CBCL Internalizing	13.80 (9.3)	19.31 (14.01)	16.80 (8.41)	28.35 (18.25)	5.74**	.001	5.91*	.024
CBCL Externalizing	9.79 (5.45)	14.48 (9.26)	14.32 (7.43)	19.38 (10.91)	5.20**	.002	3.19	.085

Note. PTSD = Posttraumatic stress disorder, CPTSD = complex posttraumatic stress disorder. CBCL internalizing = CBCL internalizing difficulties; CBCL externalizing = CBCL externalizing difficulties. *p < .05, **p < .01.

Post-hoc univariate analysis showed that children of mothers with CPTSD had significantly more internalizing difficulties compared to children of mothers with PTSD (Cohen’s *d* = 2.30) as well as healthy mothers (Cohen’s *d* = 2.86), but not compared to mothers with depression only, although this difference was large in magnitude (Cohen’s *d* = 1.86). The latter non-significant difference must be considered with caution, as this post-hoc test is underpowered, and the observed effect size is large in magnitude.

Furthermore, children of mothers with CPTSD had higher levels of externalizing difficulties, compared to children of healthy mothers (Cohen’s *d* = 2.32). Although all other group comparisons were not statistically significant, these post-hoc sub-group analyses were, as noted, underpowered. Indeed, child externalizing difficulties were much higher among children of mothers with CPTSD relative to mothers with PTSD (Cohen’s *d* = 1.24), as well as mothers with depression only (Cohen’s *d* = 1.23). Furthermore, externalizing difficulties were also higher among children of mothers with PTSD compared to healthy mothers (Cohen’s *d* = 1.08), although in contrast to CPTSD, no difference was observed between children of mothers with PTSD and mothers with depression only (Cohen’s *d* = .04, Table 5).

### 2.4. Maternal CPTSD and depression vs. PTSD and depression – differences in child outcomes

Finally, children of mothers with CPTSD and probable depression (*n* = 28) demonstrated marginally significantly higher child internalizing problems, but of a large standardized mean difference (SMD; measure of effect size), compared to children of mothers with PTSD and depression (*n* = 16) (SMD = 7.83/11.73 = .67). In contrast, no difference was observed between these comorbid sub-groups for child externalizing difficulties (SMD = 3.19/9.09 = .35). Table 6 presents the means and standard deviations and the test statistics of the comparisons.

**Table 6.** Comparisons between mothers with CPTSD and depression vs. mothers with PTSD and depression.

	CPTSD and depression *n* = 28	PTSD and depression *n* = 16	Probable diagnosis	*p*
** **			T (39)	
CBCL Internalizing	29.26 (18.52)	21.43 (8.47)	1.85	.071
CBCL Externalizing	19.50 (10.93)	16.31 (6.57)	.97	.337

Note. PTSD = Posttraumatic stress disorder, CPTSD = complex posttraumatic stress disorder, CBCL internalizing  =  CBCL internalizing difficulties; CBCL externalizing = CBCL externalizing difficulties.

## 3. Discussion

This is the first study to explore the intergenerational effect of maternal ICD-11 CPTSD on child internalizing and externalizing difficulties, while accounting for adverse child experiences post-displacement, among Eritrean asylum-seeking mothers and their preschool-aged children living in a high-risk post-displacement urban setting. First, a total of 26.0% of mothers met ICD-11 PTSD (but not CPTSD) criteria and 23.6% of mothers met ICD-11 CPTSD criteria. Due to the hierarchical taxonomic structure of ICD-11 CPTSD, all mothers with probable CPTSD also met criteria all symptom criteria for probable PTSD. Moreover, 62.2% of mothers demonstrated moderately severe or severe depression symptoms. Strikingly high rates of comorbid depression were observed among mothers with ICD-11 CPTSD (94.5%). Depression was also comorbid, but at much lower rates, among mothers with ICD-11 PTSD (48.5%). These results thus align with previously observed prevalence for DSM PTSD (52.9% to 71.1%) and depression (46% to 50%) in treatment-seeking and community mixed-gender samples in this population and post-displacement setting (Aizik-Reebs et al., 2021; Youngmann et al., 2021). The very high prevalence and comorbidity rates are clinically plausible given the high number of pre-, peri- and post-migration traumatic experiences, as well as very high rates and severity of chronic post-migration living difficulties in this population of asylum-seekers (e.g. Aizik-Reebs et al., 2021; Youngmann et al., 2021; Yuval et al., 2021).

Second, findings implicate maternal CPTSD in intergenerational transmission of trauma and stress for child internalizing and externalizing difficulties. Indeed, relative to maternal PTSD, maternal CPTSD was significantly and strongly associated with elevated child internalizing difficulties and marginally significant, although strongly, associated with child externalizing difficulties. These strikingly large effects suggest that ICD-11 CPTSD, even relative to ICD-11 PTSD, may be functionally important to elevated risk for maternal intergenerational transmission of trauma and stress to children. Notably, these effects were observed above and beyond the effects of direct child exposure to adverse experiences post-displacement. Thus, observed poor child socioemotional outcomes were not alternatively accounted for by children’s direct exposure to adverse childhood experiences. Finally, future studies may explore the mechanism(s) that implicate CPTSD in intergenerational transmission of trauma. For example, it may be that the intergenerational toxicity of CPTSD DSO symptoms, which are not part of the narrower ICD-11 PTSD syndrome, may be a marker of more severe psychopathology than PTSD. For example, parents with CPTSD typically experience difficulties in sustaining relationships and feeling close to others (WHO, 2018), which could be detrimental to the parent-child relationship. Indeed, insecure attachment has been identified as both a contributing factor to a CPTSD diagnosis (Karatzias et al., 2018) as well as a risk factor for poor child outcomes (e.g. Cooke et al., 2019). Moreover, parents who suffer from CPTSD struggle with emotion regulation, which can result in anger outbursts and harsher parenting styles (Bryant et al., 2018; Sim et al., 2018). Finally, the nearly universal expression of depression among mothers with CPTSD is a likely contributor to maladaptive child outcomes (e.g. East et al., 2018).

Third, in light of the striking pattern of comorbidity between depression and CPTSD, through post-hoc exploratory analyses, we sought to begin to explore whether CPTSD, depression or their combined effect may be largely responsible for observed intergenerational transmission effects. Importantly, relative to maternal PTSD and depression, maternal CPTSD and depression were significantly and strongly associated with elevated child internalizing difficulties, but not greater externalizing symptoms. In addition, relative to maternal unimorbid depression, maternal CPTSD and depression were not significantly associated with elevated child internalizing or externalizing symptoms, although these (null) associations were large and moderate in magnitude, respectively. Though post-hoc and underpowered, these analyses indicate that though strongly linked to comorbid maternal depression, CPTSD appears to confer a significant risk for intergenerational transmission of trauma and stress for child internalizing and externalizing symptoms. The effects appear particularly more robust and large for internalizing symptoms.

Notably, although the high comorbidity between CPTSD and depression has been previously documented (e.g. Hyland, Shevlin, Elklit, et al., 2017), the present study and findings are, to the best of our knowledge, the first seeking to understand their complex inter-relations with respect to intergenerational transmission in the context of forced displacement. In so far as CPTSD occurs in the absence of depression, sampling of future studies may be designed to dissociate these forms of trauma- and stress-related symptomatology from one another concerning child outcomes. In so far as CPTSD and depression may not be nosologically comorbid but rather systemically or functionally indistinguishable, such findings may have taxonomic implications for classifying and diagnosing these conditions broadly or among forcibly displaced people more specifically. We believe that this is an important frontier for ongoing critical research with implications for intergenerational transmission, for prevention and intervention of child outcomes linked to maternal trauma and stress, as well as for taxonomy and psychiatric nosology.

Briefly, it is notable that present findings are aligned with previous evidence linking DSM-IV, DSM-5 and ICD-10 PTSD and maladaptive child outcomes among refugee- and asylum-seeking populations (e.g. Dalgaard et al., 2016; East et al., 2018; Eruyar et al., 2018; van Ee et al., 2012). This is important because previous taxonomic and diagnostic instantiations of PTSD systematically ad-mixed phenotypically heterogeneous PTSD fear and CPTSD DSO symptomatology (Hyland, Shevlin, Brewin, et al., 2017). The present findings thus suggest that previous findings linking PTSD to intergenerational transmission among forcibly displaced families (Flanagan et al., 2020; Sangalang & Vang, 2017) may have been largely accounted for by the sub-group of mothers struggling with CPTSD symptomatology (Bachem et al., 2021).

Study findings may have several clinical implications for forcibly displaced mothers struggling to cope and adapt with trauma and chronic stress, as well as for their children’s socioemotional development post-displacement. First and foremost, systems of care should be aware of the very high risk for socioemotional developmental problems among children of forcibly displaced mothers struggling with CPTSD. Notably, even in the absence of CPTSD, children of forcibly displaced people, often living in under-resourced high-risk post-displacement settings, are vulnerable to socioemotional developmental problems (Flanagan et al., 2020; Vossoughi et al., 2018). The present findings indicate that maternal CPTSD radically amplifies this already elevated risk. The consequences of internalizing and externalizing difficulties for children are significant and long-term, and include, for example, academic underachievement (Shi & Ettekal, 2021), experiences of peer victimization (van Lier et al., 2012), and lower perceived physical health (Arslan et al., 2021).

Targeted prevention and early intervention strategies to attenuate intergenerational transmission should focus on maternal psychopathology (CPTSD, PTSD, depression) as well as relevant relational mechanisms. First meta-analytic evidence suggests that for addressing the CPTSD symptom spectrum, multicomponent interventions including trauma-focused strategies along with interventions to foster emotion regulation and distress tolerance show the best effects (Karatzias et al., 2019). Flexibly applied additional modules focusing on specific problems of concern, such as comorbidities, could be combined (Karatzias & Cloitre, 2019). It has been stressed that treating maternal trauma-related disorders is imperative, already during the perinatal period, in order to prevent maternal mental and physical health problems following childbirth (Nillni et al., 2018). Moreover, behavioural parenting training (see Gillespie et al., 2022, for a review) may be critically important to ensure that children of forcibly displaced people are afforded the opportunity to thrive and flourish. Such parent training should be socio-culturally adapted, trauma-sensitive, and focus on enhancing parenting skills, promoting attachment, and reducing parenting stress. Forcibly displaced parents may thus be supported in providing a safe and nurturing environment for their children that can help attenuate pathogenic developmental processes linked to attachment or emotion regulation.

Considering the global scale of forced displacement, and the estimated population-prevalence of CPTSD in increasingly common unstable high-risk post-displacement settings, the global health implications of effective clinical care to attenuate intergenerational transmission of trauma and stress may prove significant to forcibly displaced- and their host nation- communities. Finally, it is critical that to effectively attenuate and prevent intergenerational transmission of trauma post-displacement, efforts and resources must be invested beyond early identification and intervention. Indeed, beyond mental health care, policies linked to re-settlement, residential status, socioeconomic mobility, and restorative justice are critical to attenuate CPTSD, depression and related conditions among forcibly displaced parents to begin with as well as to attenuate risk for intergenerational transmission to their children (Reed et al., 2012).

The present study has several limitations that may qualify findings and inform future studies. First, because the structured International Trauma Interview (ITI; Roberts et al., 2019) was not yet available at the time of the study, measurement relied on well-established self-report questionnaires. Moreover, clinician-rated instruments, such as the Hamilton (Hamilton, 1967), do not have socio-cultural generalizability to diverse refugee populations and may lead to diagnostic bias and low inter-rater reliability. Thus, we selected well-established self-report measures that have been previously studied among refugees and asylum-seekers, and that underwent extensive translation and back-translation as well as cognitive interviewing with members of the studied East African refugee community. Notably, our previous work over the past decade with this population (AB) has indicated that self-report may produce more valid reports and estimates of highly stigmatized outcomes (e.g. mental health) than diagnostic or unstructured interviews due to strong social demand characteristics (Aizik-Reebs et al., 2022). However, the ITQ and CBCL were translated for the current study, showing good internal consistency, but have not separately been psychometrically validated. Notably, these particular measures have been widely translated and studied in dozens of language and population groups around the world. Second, it is possible that some of the association between self-reported maternal mental health and mother’s report on child behaviour problems may be due to shared method and informant variance. However, the differential pattern of associations between maternal psychopathology and child outcomes in the present data suggests that this is not likely a plausible threat to the internal validity of study findings. Moreover, this same measurement limitation is common to hundreds of studies using the CBCL parent-report forms and a logistical constraint in community-embedded studies among refugees and asylum-seekers in high-risk settings wherein valid multi-informant sampling and measurement is not feasible. Nevertheless, it is important that future studies replicate and extend the present findings with additional methods, including independent observational data on child behaviour. Third, the cross-sectional nature of the data does not permit strong causal interpretation of the findings. Future experimental, randomized control intervention, and longitudinal designs are important to rigorously test the present findings.

In summary, the present study is, to the best of our knowledge, the first to investigate and document the role of maternal ICD-11 CPTSD and comorbid depression, above and beyond direct adverse child experiences, for intergenerational transmission of trauma on child socioemotional development among forcibly displaced mothers and their preschool-aged children living in a high-risk post-displacement urban setting. Findings implicate CPTSD and comorbid depression in elevated child internalizing and externalizing outcomes, although effects are more robust and larger for internalizing symptoms. Findings may have important implications for ongoing study to advance understanding of intergenerational transmission of trauma and stress among asylum-seeking mothers as well as for guiding clinical assessment, prevention, and intervention to attenuate poor socioemotional development outcomes among children in post-displacement settings.

## Supplementary Material

Supplementary_Material.docx

## Data

The data for this manuscript is stored on Open Science Framework: https://osf.io/q9b3e/?view_only = 05c371ddc8974db9819c657b344b8f12.
