# Supplementary material for: Complex posttraumatic stress disorder in intergenerational trauma transmission among Eritrean asylum-seeking mother-child dyads
Source: Eur J Psychotraumatol. 2024 Jan 8;15(1):2300588. doi: 10.1080/20008066.2023.2300588 (PMC10776052; doi:10.1080/20008066.2023.2300588)
Supplement: Supplementary_Material.docx [file ZEPT_A_2300588_SM1178.docx]

**Supplementary Materials**

Complex posttraumatic stress disorder in intergenerational trauma transmission among Eritrean asylum-seeking mother-child dyads

**Additional comparisons**

Moderate-sized but not statistically significant differences were found between children whose mothers had PTSD (some of whom also had depression) and children of healthy mothers (Cohen's d = 0.58), and between children of mothers with PTSD, some of whom also had depression, and children of mothers with depression only (Cohen's d = 0.52).

**Sensitivity analyses**

We conducted two sensitivity analyses testing the implication of maternal PTSD and CPTSD on a child's difficulties. In the first model, we compared maternal PTSD and CPTSD, while controlling their shared variance, so that the effect of CPTSD on the child's difficulties is "cleaned" from PTSD, yielding the unique effect of DSO, or the bigger than its sum CPTSD. An effect of PTSD only demonstrates that PTSD only has a main effect. In the second model, we compared maternal PTSD and CPTSD DSO in their effects on the child's difficulties.

In the model testing for the associations between maternal PTSD and CPTSD (figure SM1: chi-square (df=2) =12.88 *p* = .002 CFI = .961 RMSEA = .20) it can be seen that while higher severity of CPTSD symptoms (overall PTSD+DSO symptoms) was associated with more severe child difficulties, that are both internalizing and externalizing, PTSD was not associated with the child's difficulties, controlling for the effect the life events of the child have for his/her difficulties.

In the model testing for the associations between maternal PTSD and DSO CPTSD (figure SM2: chi-square (df=2) =12.32 *p* = .002 CFI = .961 RMSEA = .20), it can be seen that while higher severity of DSO CPTSD symptoms was associated with more severe child difficulties, that are both internalizing and externalizing, PTSD was only associated with internalizing difficulties, but not with externalizing difficulties, controlling for the effect the life events of the child have for his/her difficulties.

**Summary of sensitivity analysis**

The severity of ICD-11 CPTSD, relative to the narrower ICD-11 PTSD, was significant concerning child internalizing and externalizing difficulties. Furthermore, the examination of ICD-11 DSO vs. PTSD revealed that the association between maternal DSO and both child’s difficulties were significant, but the association between PTSD and internalizing difficulties was also significant.


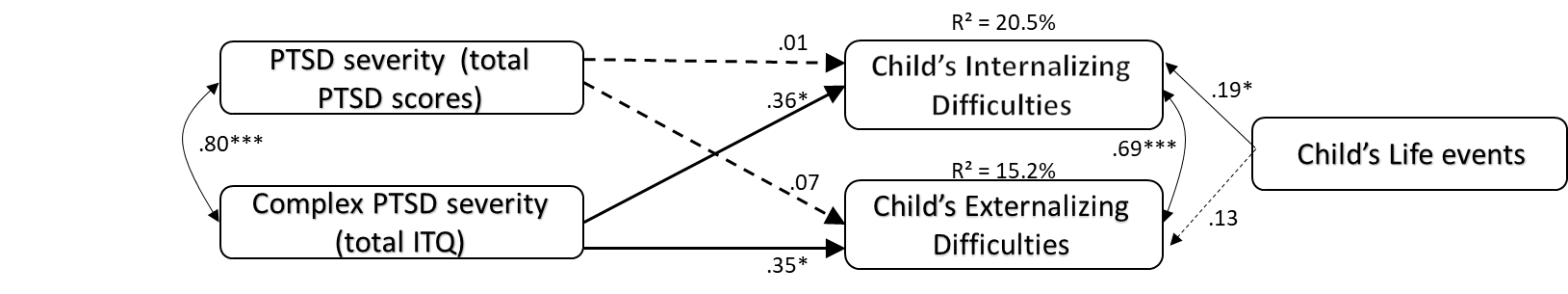


**Figure SM1.**

Standardized coefficients of the path analysis predicting child's difficulties by maternal PTSD and CPTSD (continuous scores)


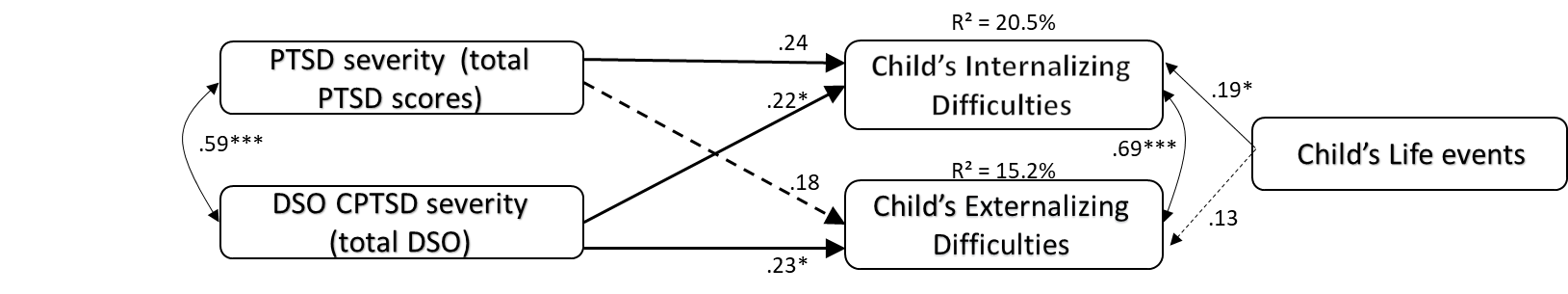


**Figure SM2.**

Standardized coefficients of the path analysis predicting child's difficulties by maternal PTSD and DSO CPTSD (continuous scores)
